# Screening of the Risk of Eating Disorders Among Medical Students in the MENA region and Its Associated Factors: A Multinational Cross‐Sectional Study

**DOI:** 10.1002/brb3.70417

**Published:** 2025-03-18

**Authors:** Siwar Belhaj Salem, Fatima Ezzahraa El Idrissi, Ahmed Fikry mohamed, Husam Khrais, Rawan Elwalid Jad, Umair Ul Haq, Jaafer Ammar Jouini, Ramla Mohamed Farah Roble, Ronahy Haidar, Fatma Zaouali, Abdullah Janem, Hossam Salameh, Ruzan Jamaleddin, Alaa Mohamed Elsayed, Fidha Hussain, Yasmine Adel Mohammed, Abeer Hagali, Zeyad Yassin, Shahd Almansour, Farah Bahsas, Dana AlMahder, Muhammad Shoaib Khan

**Affiliations:** ^1^ Department of Family Medicine Faculty of Medicine of Monastir Monastir Tunisia; ^2^ Faculty of Medicine Pharmacy and Dental Medicine of Fez ‐ Sidi Mohammed Ben Abdellah University Fez Morocco; ^3^ Faculty of Medicine South Valley University Qena Egypt; ^4^ Faculty of Medicine at Hashemite University Zarqa Jordan; ^5^ International Medical University Kuala Lumpur Malaysia; ^6^ Bannu Medical College Bannu Pakistan; ^7^ Faculty of Medicine Damascus University Damascus Syria; ^8^ School of Medicine Ahfad University for Women Omdurman Sudan; ^9^ Faculty of Medicine University of Aleppo Aleppo Syria; ^10^ Emergency Department Haj Ali Soua Regional Hospital Monastir Tunisia; ^11^ Department of Medicine, Faculty of Medicine and Health Sciences An‐Najah National University Nablus Palestine; ^12^ Faculty of Medicine Menofia University Menofia Egypt; ^13^ Department of Oncology Northampton General Hospital Northampton UK; ^14^ Department of Pharmacology, Faculty of Medicine Assiut University Assiut Egypt; ^15^ Faculty of Medicine University of Medical Sciences and Technology Khartoum Sudan; ^16^ Faculty of Medicine Alazhar University Cairo Egypt; ^17^ Faculty of Medicine Homs University Homs Syria

**Keywords:** anorexia nervosa, bulimia nervosa, feeding and eating disorders, medical students, MENA, prevalence, underweight

## Abstract

**Background:**

Eating disorders (EDs) are a group of mental diseases marked by disrupted eating behaviors, and are associated with several factors. Medical students are highly exposed to this mental disorder with a prevalence of 17.35% in 2022.

**Objective:**

To evaluate the risk of EDs and its associated factors among medical students in the Middle East and North Africa (MENA) region.

**Methods:**

A web‐based cross‐sectional study was conducted among medical students in the MENA region during the months of June and July 2024. The primary data collection instrument was a comprehensive questionnaire that contained the Eating Attitudes Test (EAT‐26) and sociodemographic and clinical features and designed using Google Forms and distributed via social media platforms.

**Results:**

The total number of participants was 5061. The mean age in our population was 22.58 ± 3.27. Our population's average EAT‐26 score was 13.87 ± 10.7, with ranges varying from 0 to 72. Based on their EAT‐26 scores being 20 or above, 1254 people (24.8%) were deemed to be at risk of EDs. Among the participants, 8% were underweight. The multivariable logistic regression model revealed several eating disorder risk factors such as T1DM, schizophrenia, autism, female gender, IBD, and daily exposure to thin body ideal. Regular sports practice and weight satisfaction were protective factors.

**Conclusion:**

There exists a higher prevalence of individuals at risk for the development of EDs in the MENA region especially females, students with comorbidities, and those having conflictual relationships with their parents. Regular sports practice and weight satisfaction are protective factors.

## Graphical abstract

Our study underscores the heightened susceptibility to eating disorders among medical students, along with the associated risk factors. Students with comorbid conditions exhibit an increased risk. Conversely, weight satisfaction and regular participation in physical activities are protective factors.

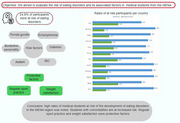

**Abbreviations**
ADHD: Attention deficit hyperactivity disorder
AN: Anorexia nervosa
ASD: Autism spectrum disorder
BED: Binge eating disorder
BN: Bulimia nervosa
BMI: Body mass index
EAT‐26: Eating Attitudes Test 26
EDs: eating disorders
GI: Gastrointestinal
IBD: Irritable bowel disease
KSA: Kingdom of Saudi Arabia
MENA: Middle East and North Africa
MDD: Major depressive disorder
OCD: Obsessive‐compulsive disorder
PTSD: Post‐traumatic stress disorder
T1DM: Type 1 diabetes mellitus
UAE: United Arab Emirates

## 1 Introduction

Eating disorders (EDs) are a group of mental illnesses characterized by a dysfunction in eating behaviors or weight‐control practices. Several types of EDs exist, including anorexia nervosa (AN), bulimia nervosa (BN), binge eating disorder (BED), rumination disorder, pica, and avoidant restrictive food intake disorder (ARFID). AN is characterized by an excessive fear of weight gain or a distorted body image, or a combination of both, which prompts significant dietary restrictions or other weight loss behaviors. BN is associated with binge episodes, and attempts to counteract these binge eating episodes, which may include practices such as purging, excessive physical activity, fasting, or inadequate insulin administration in individuals with Type 1 diabetes with an over‐evaluation of the importance of weight and/or shape. BED is characterized by recurrent episodes of excessive food consumption, during which individuals exhibit a diminished sense of control over their eating and are not associated with significant compensatory behaviors (Feng et al. 2023). The pathogenesis of EDs involves many factors. First, predisposing factors have been identified such as genetic vulnerability, temperamental traits, and childhood traumatic experiences. Second, the aggravating factors include the environmental context at the time of onset, and lastly, the stabilizing factors which incorporate the secondary aspects of the illness like brain adaptation induced by malnutrition, social isolation, and changes in the environment (Solmi et al. 2024). Epidemiological studies confirmed that the risk of AN increases in young individuals worldwide. IN addition, the prevalence rate of AN is approximately 4% among females compared to 0.3% among males. On the other hand, BN incidence tends to decrease with age, and incidence among females and males is 3% and 1% respectively (van Eeden et al. 2021; Galmiche et al. 2019). Therefore, it is thought that the etiology of EDs is sex‐related due to their female predominance (Feng et al. 2023).

Medical students frequently encounter this mental disorder as a result of heightened levels of stress, depression, and anxiety (Dyrbye et al. 2006; Ishak et al. 2013; Elzubeir et al. 2010). Cultural, religious, and demographic factors have a substantial impact on the prevalence of EDs among medical students in the MENA region. Social media and modern beauty contribute to increased body dissatisfaction. Traditional gender stereotypes and higher academic requirements in medical education aggravate the hazards (Elzubeir et al. 2010). A higher prevalence of restrictive eating behaviors is seen among females due to higher heritability compared to males. Diabetes is commonly associated with EDs which may lead to poor glycemic control as a result of insulin restrictions. Psychological illnesses, due to genetic associations, are highly correlated to EDs. Binge‐type EDs (BN and BED) are genetically associated with ADHD. Conversely, AN is in genetic correlation with OCD, MDD, suicidality, schizophrenia, and autism (Elzubeir et al. 2010; Barakat et al. 2023; Kussman and Choo 2024). Other risk factors were identified, including childhood trauma, previous exposure to a stressful event, and perfectionism (Barakat et al. 2023; Cascino et al. 2023).

The frequency of mental health disorders in medical students, a category extremely prone to mental health disorders, has increased from 10.4% in 2019 to 17.35% in 2022 (Fekih‐Romdhane et al. 2022; Jahrami et al. 2019). Therefore, comprehensive screening programs are the most effective techniques for preventing serious complications of advanced EDs. Regular screening of EDs via questionnaires may aid in the early detection of the illness and referral to a qualified health professional in order to confirm the diagnosis and outline the appropriate treatment plan. Furthermore, a better understanding of its symptoms and presentation among youngsters can aid in both primary and secondary prevention stressing the importance of this study (Iyer and Shriraam 2021).

There have been limited studies focusing on addressing the total burden of EDs in medical students of the MENA region, making it even more difficult for students to seek help or receive adequate support. Thus, the identification of ED‐associated factors is crucial in order to establish culturally appropriate approaches to ED prevention and intervention. Research in this area can help raise awareness, improve training for healthcare professionals, and lead to better mental health resources within medical schools.

The objectives of our studies are:

1. Evaluate the risk of EDs among medical students in the MENA region.
2. Identify and evaluate the factors associated with EDs in this population.

## 2 Methods

### 2.1 Study Design

A web‐based cross‐sectional study was conducted to evaluate the risk of EDs and its associated factors among medical students drawn from countries in the Middle East and North Africa (MENA), as per the World Bank classification. The survey included twelve countries (Palestine, Egypt, Syria, Tunisia, Morocco, Sudan, United Arab Emirates, Jordan, Algeria, Libya, Saudi Arabia, and Pakistan) during the month of June 2024. The primary instrument for data collection was a comprehensive questionnaire that included the Eating Attitudes Test (EAT‐26) (Garner et al. 1982).

### 2.2 Participants

The inclusion criteria were as follows:

● Participants aged 18 years and above.
● Undergraduate medical students currently enrolled in their respective institutions, including students from the first academic year through the sixth academic year.
● Interns were included, which designates undergraduate students during their first year of supervised clinical training, where they acquire practical experience in patient care (Rosen and Gross 1995).
● Medical residents are defined as graduate students who have completed medical school and are undergoing specialized training in a specific medical field, under the guidance of attending physicians (Peterson et al. 2023).
● Must be from a MENA country according to the World Bank classification.

### 2.3 Questionnaire Methods

The questionnaire was designed using Google Forms. In order to uphold ethical standards and obtain reliable results, we utilized Facebook groups, X, and WhatsApp for the distribution of our questionnaire, recognizing that these platforms are prevalent among our target demographic. We aimed to minimize any potential bias by disseminating the survey in a neutral and unobtrusive fashion. This approach allowed participants to engage voluntarily without any undue pressure to respond. We employed this method because we did not have access to other sampling methods, such as using student emails or a database, and random sampling was not feasible. Participation in the survey was voluntary and electronic informed consent was obtained from all participants. Participants had the right to withdraw from the questionnaire at any time. The estimated time to complete the questionnaire was approximately 10 min. All responses were treated anonymously and confidentially; participants’ names were kept in a password‐protected database by the investigators and were only associated with a study identification number. Key variables were selected based on existing literature on EDs among medical students. Demographic variables, specifically age, gender, and academic year, were incorporated to explore potential associations. Self‐reported measurements of weight and height were utilized to compute Body Mass Index (BMI). Furthermore, lifestyle factors such as physical activity levels and sleep patterns, as reported by participants, were evaluated due to their recognized relationships with disordered eating behaviors. In addition, psychological comorbidities, exposure to societal standards of thinness, satisfaction with body weight, and food insecurity were included as self‐reported conditions to evaluate their potential role in eating behaviors.

For the assessment of ED risk, the EAT‐26 has been reproduced with permission. This questionnaire is a validated tool for the screening of ED symptoms (Garner et al. 1982).

The EAT‐26 consists of 26 questions, each with six response options:

For questions from 1 to 25: Always (3), usually (2), often (1), sometimes, rarely or never (0).
For question number 26: Never (3), rarely (2), sometimes (1), always, usually or often (0).

A total score can range from 0 to 78 based on the answers to all questions. A score of 20 or higher indicates possible disrupted eating behaviors (Garner et al. 1982). The cutoff score of 20 was selected based on its previous application in research aimed at identifying individuals who may be at risk for disordered eating behaviors (Garner et al. 1982; Rosen and Gross 1995). This threshold has been shown to balance sensitivity and specificity effectively, providing a reliable screening tool for disordered eating attitudes in both clinical and non‐clinical populations, including medical students.

The participants completed the EAT‐26 in their preferred language, selecting either Arabic or English. The Arabic version of the EAT‐26 demonstrated good internal consistency (Cronbach's *α* = 0.895) and accounted for 60.07% of the variability in disordered eating behaviors within a sampled population from Lebanon. However, sensitivity and specificity estimates were not provided (Haddad et al. 2021).

### 2.4 Sample Size

The sample size was determined using OpenEpi's sample size calculator for proportions. With a prevalence of 17.35%, a confidence level of 95%, and a 5% margin of error, the initial sample size required was 220 participants. To account for 20% data loss, the final target was adjusted to 275 participants.

### 2.5 Ethical Considerations

Ethical approval was obtained from the Director of Research and Medical Journal at Bannu Medical College, Pakistan, on June 6, 2024. All methods were performed in accordance with the relevant guidelines and regulations.

### 2.6 Statistical Analysis

Data were analyzed using IBM SPSS Statistics software 28.0.

Descriptive statistics were computed to summarize the demographic and clinical characteristics of the participants. The normality of the distribution of continuous variables was assessed using the Kolmogorov–Smirnov test. Continuous variables were normally distributed. Therefore, mean and standard deviations (SD) were used. Frequencies and percentages were used for categorical variables. For inferential statistics, the Chi‐square test was used to analyze the differences in proportions between categorical variables. We used a binary outcome variable based on the EAT‐26 score, where participants were classified as either exhibiting significant disordered eating symptoms (EAT‐26 score greater than 20) or not. To quantify the participation of independent variables in the outcome of interest, a multi‐variate analysis through logistic regression was performed after satisfying all the conditions. The variables that presented a significance level of up to 20% in the bivariate test were included. The null hypothesis was rejected for an alpha value less than 0.05.

### 2.7 Bias

Participants were selected via convenience sampling to ensure practicality, with efforts made to encompass diverse representation from medical schools across the MENA region. Confidentiality and anonymity were guaranteed to foster truthful responses and clear communication about the study promoted participation. Standardized elements from the EAT‐26 and behavioral features associated with EDs were employed to minimize measurement bias. Specific, clear questions focused on recent behaviors were used to reduce recall bias. Data analysts were blinded to participant identities, and standardized training and protocols ensured consistent data collection.

## 3 Results

**FIGURE 1:**
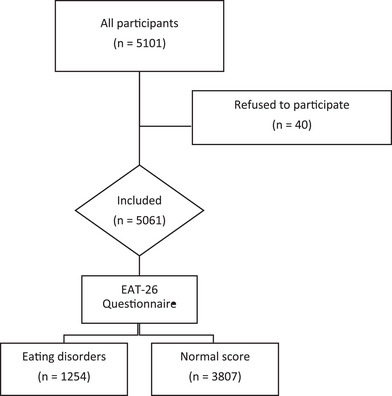
Flowchart.

In this study, a total of 5061 participants were included as shown in Figure 1.

### 3.1 Sociodemographic Features

**FIGURE 2:**
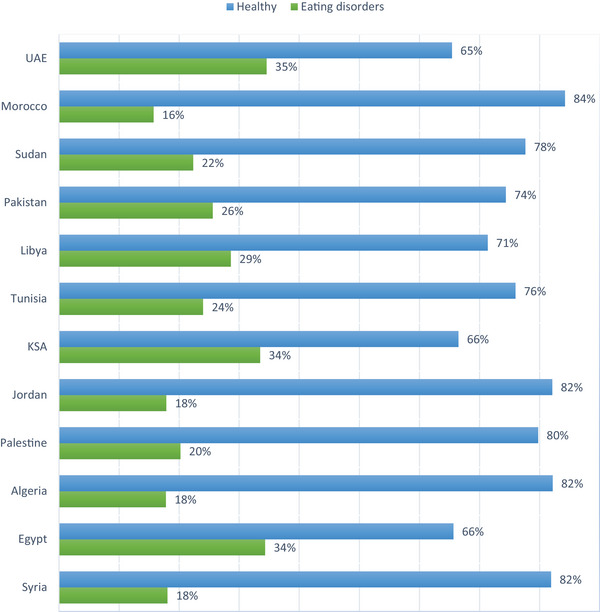
Prevalence of eating disorders per country.

The sociodemographic features of our population are detailed in Table 1. The mean age in our population was 22.58 ± 3.27, varying from 18 to 36 years. The most represented age group was between 21 and 25 years, with 2891 individuals forming 57.1% of the sample. A female predominance was noted, consisting of 3296 responses (65.1%). Our population included 12 countries as represented in Figure 2. Egypt has the largest sample size with 1165 participants (23%), followed by Syria, with 902 participants forming 17.8% of the population. Third‐ and Fourth‐year students were among the highest, with 1002 (19.8%) students in the former and 936 (18.5%) in the latter. The overwhelming majority were single, forming 92.8% of the responses. Responders were non‐smokers in 90.7%.

**TABLE 1:** Sociodemographic features of study participants.

Variable	Category	Frequency (*n*)	Percentage (%)
**Age**	18–20 years	1387	27.4
	21–25 years	2891	57.1
	26–30 years	612	12.1
	31–36 years	171	3.4
**Gender**	Female	3296	65.1
	Male	1765	34.9
**Academic status**	1st year	656	13.0
	2nd year	600	11.9
	3rd year	1002	19.8
	4th year	936	18.5
	5th year	567	11.2
	6th year	294	5.8
	Intern	456	9.0
	Resident	550	10.9
**Marital status**	Single	4697	92.8
	Married	345	6.8
	Widowed	5	0.1
	Divorced	14	0.3
**Smoking**	No	4592	90.7
	Yes	469	9.3
**Ethylism**	No	4907	97.0
	Yes	154	3.0
**Regular sport practice**	No	3793	74.9
	Yes	1268	25.1

### 3.2 Risk Factors of ED

Identified risk factors of EDs are summarized in Table 2. The rate of participants with presumed Type 1 diabetes mellitus was 2.4%. The rate of individuals who reported having a past history of irritable bowel disease (IBD), self‐reported sleeping disorders, and autism were 14.1%, 14.2%, and 1.7% respectively. Forty‐six percent of participants reported being satisfied with their weight. Exposure to the “thin body” ideal 32.1% of participants answered daily, followed by weekly 17.4%.

**TABLE 2:** Identified risk factors of eating disorders.

Variable	Category	Frequency (*n*)	Percent (%)
**T1DM**	No	4939	97.6
	Yes	122	2.4
**IBD**	No	4345	85.9
	Yes	716	14.1
**Autism**	No	4974	98.3
	Yes	87	1.7
**Sleeping disorders**	No	4342	85.8
	Yes	719	14.2
**Hours of sleep**	< 7 h	1643	32.5
	7–9 h	2918	57.7
	> 9 h	500	9.9
**Do your parents push you to eat?**	No	3037	60.0
	Yes	2024	40.0
**Past suicide attempts**	No	4596	90.8
	Yes	465	9.2
**Weight satisfaction**	No	2716	53.7
	Yes	2345	46.3
**Thin body ideal exposure**	Daily	1625	32.1
	Weekly	883	17.4
	Monthly	636	12.6
	Yearly	274	5.4
	Rarely	878	17.3
	Never	765	15.1
**Food insecurities**	No	4034	79.7
	Yes	1027	20.3

### 3.3 Psychological Comorbidities

Participants' psychological comorbidities were reported in Table 3. Social anxiety disorder was the most frequently self‐reported psychiatric condition (12%). Second in line was OCD with 7%, followed by MDD which was 6.2%. As for suicidal attempts, 9.2% affirmed attempting this act in the past.

**TABLE 3:** Psychological comorbidities.

Variable	Category	Frequency (*n*)	Percent (%)
**Post‐traumatic stress disorder (PTSD)**	No	4766	94.2
	Yes	295	5.8
**Obsessive compulsive disorder (OCD)**	No	4709	93.0
	Yes	352	7.0
**Social anxiety disorder**	No	4456	88.0
	Yes	605	12.0
**Bipolar disorder**	No	4983	98.5
	Yes	78	1.5
**Schizophrenia**	No	5018	99.2
	Yes	43	0.8
**Major depressive disorder (MDD)**	No	4745	93.8
	Yes	316	6.2
**Borderline personality disorder**	No	4962	98.0
	Yes	99	2.0
**Perfectionism**	No	4762	94.1
	Yes	299	5.9
**Obsession**	No	4914	97.1
	Yes	147	2.9

### 3.4 Symptoms of EDs

In this section of the survey, we asked participants about whether they have a past history of ED and its type, the frequency, and severity of ED symptoms if they exist. Our findings are reported in Table 4. Among our participants, 19.8% reported having a history of EDs, of which ARFID and AN were the most frequent, 10.5% and 10.4%. When asked about the frequency of their symptoms, participants answered with “monthly” in 17.5%, and “weekly” in 14.6%. When asked about the severity of their symptoms, 51.3% answered “mild”.

**TABLE 4:** Eating disorders history and symptoms.

Variable	Category	Frequency (*n*)	Percent (%)
**History of ED**	No	4060	80.2
	Yes	1001	19.8
**Type of ED**	Anorexia nervosa	527	10.4
	Bulimia nervosa	164	3.2
	Binge eating disorder	480	9.5
	Avoidant or restrictive food intake disorder	531	10.5
	Other	1546	30.5
	Total answered	3248	64.2
	Unanswered	1813	35.8
**Frequency of ED symptoms**	Daily	465	12.0
	Weekly	566	14.6
	Monthly	681	17.5
	Yearly	250	6.4
	Rarely	1006	25.9
	Never	918	23.6
	Total answered	3886	76.8
	Unanswered	1175	23.2
**Severity of the symptoms**	Mild	1766	51.3
	Moderate	1295	37.6
	Severe	345	10
	Very severe	38	1.1

### 3.5 Body Mass Index (BMI)

The mean BMI, as calculated from self‐reported weight and height, was 24.1 ± 4.8 with extremes ranging from 12.1 to 69.6 kg/m^2^. Eight percent were underweighted (BMI under 18.5 kg/m^2^) (As shown in Table 5).

**TABLE 5:** Repartition of participants according to their BMI.

Category	Frequency (*n*)	Percent
**Underweight**	405	8.2
**Healthy range**	2654	53.6
**Overweight**	1377	27.8
**Obesity**	491	9.9
**Severe obesity**	29	0.6

### 3.6 The EAT‐26 Questionnaire

#### 3.6.1 Screening of the Risk of Developing EDs

**FIGURE 3:**
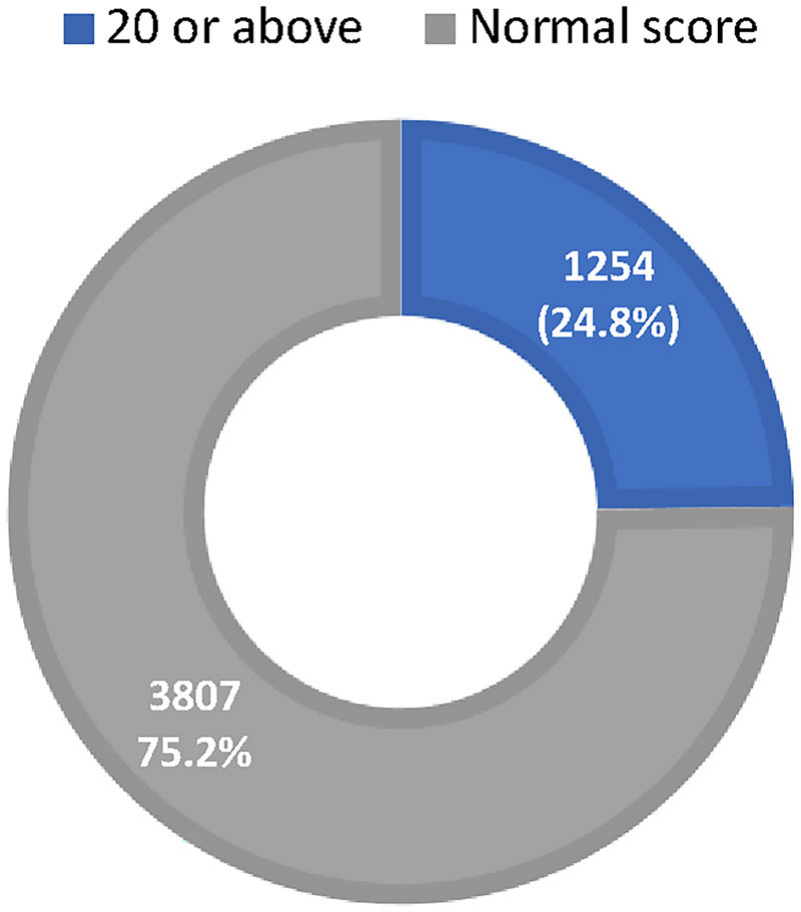
Eat‐26 score distribution.

The mean EAT‐26 score in our population was 13.87 ± 10.7 with ranges varying from 0 to 72. Out of the 5061 responses obtained, 1254 individuals (constituting 24.8% of all participants) might be at risk of EDs based on their eating attitude test (EAT‐26) scores being 20 or above (as illustrated in Figure 3). The highest rates were observed among students from UAE (35%), Egypt, and Saudi Arabia (35%) (as shown in Figure 3).

#### 3.6.2 Behavioral Questions

Participants' responses in this section are illustrated in Table 6.

**TABLE 6:** Behavioral questions.

In the past 6 months have you:	Never	Once a month or less	2–3 times a month	Once a week	2–6 times a Week	Once a day or more
**Gone on eating binges where you feel that you may not be able to stop?**	2387 (47.2)	1382 (27.3)	598 (11.8)	374 (7.4)	191 (3.8)	129 (2.5)
**Ever made yourself sick (vomited) to control your weight or shape?**	4303 (85)	298 (5.9)	185 (3.7)	147 (2.9)	73 (1.4)	55 (1.1)
**Ever used laxatives, diet pills, or diuretics (water pills) to control your weight or shape?**	4181 (82.6)	346 (6.8)	164 (3.2)	159 (3.1)	128 (2.5)	83 (1.6)
**Exercised more than 60 min a day to lose or to control your weight?**	2881 (56.9)	654 (12.9)	396 (7.8)	415 (8.2)	594 (11.7)	121 (2.4)
**Lost 20 pounds or more in the past 6 months**	**Yes**		776 (15.3)			
	**No**		4285 (84.7)			
**Have you ever been treated for an eating disorder?**	**Yes**		529 (10.5)			
	**No**		4532 (89.5)			

### 3.7 EDs Associated Factors

Our results showed that 26.2% of female participants tended to be at risk of developing EDs, while the rate was 22% among male students (Table 7). We found that 63.1% of students with self‐reported T1DM had an EAT‐26 score above 20, while 46.1% of participants with self‐reported IBD and 65.5% with self‐reported autism reported a history of an eating disorder. Furthermore, 40.5% of participants with self‐reported sleeping disorders were deemed at risk of having ED.

**TABLE 7:** Differences between students according to their EAT‐26 score and chi‐square test results.

Variables	Categories	EAT‐26 score at or above 20 *n* (%)		*p* value
		Yes	No	
Gender	Female	865 (17.1)	2431 (48.0)	0.001
	Male	389 (7.7)	1376 (27.2)	
Smoking	No	1125 (22.2)	3467 (68.5)	0.16
	Yes	129 (2.5)	340 (6.7)	
Ethylism	No	1214 (24)	3693 (73)	0.705
	Yes	40 (0.8)	114 (2.3)	
Regular sport practice	No	846 (16.7)	2947 (58.2)	0.000
	Yes	408 (8.1)	860 (17)	
T1DM	No	1177 (23.3)	3762 (74.3)	0.000
	Yes	77 (1.5)	45 (0.9)	
IBD	No	924 (18.3)	3421 (67.6)	0.000
	Yes	330 (6.5)	386 (7.6)	
Do your parents push you to eat?	No	699 (13.8)	2339 (46.2)	0.000
	Yes	556 (11.0)	1468 (29.0)	
PTSD	No	1140 (22.5)	3626 (71.6)	0.000
	Yes	114 (2.3)	181 (3.6)	
OCS	No	1125 (22.2)	3584 (70.8)	0.000
	Yes	129 (2.5)	223 (4.4)	
Social anxiety disorder	No	1023 (20.2)	3433 (67.8)	0.000
	Yes	231 (4.6)	374 (7.4)	
Bipolar disorder	No	1212 (23.9)	3771 (74.5)	0.000
	Yes	42 (0.8)	36 (0.7)	
Schizophrenia	No	1226 (24.2)	3792 (74.9)	0.000
	Yes	28 (0.6)	15 (0.3)	
MDD	No	1116 (22.1)	3629 (71.7)	0.000
	Yes	138 (2.7)	178 (3.5)	
Borderline personality disorder	No	1204 (23.8)	3758 (74.3)	0.000
	Yes	50 (1.0)	49 (1.0)	
Perfectionism	No	1136 (22.4)	3626 (71.6)	0.000
	Yes	118 (2.3)	181 (3.6)	
Obsession	No	1199 (23.7)	3715 (73.4)	0.001
	Yes	55 (1.1)	92 (1.8)	
Impulsiveness	No	1196 (23.6)	3698 (73.1)	0.003
	Yes	58 (1.1)	109 (2.2)	
Weight satisfaction	No	826 (16.3)	1890 (37.3)	0.000
	Yes	428 (8.5)	1917 (37.9)	
Food insecurities	No	871 (17.2)	3163 (62.5)	0.000
	Yes	383 (7.6)	644 (12.7)	
Sleep disorders	No	963 (19.0)	3379 (66.8)	0.000
	Yes	291 (5.7)	428 (8.5)	
Autism	No	1197 (23.7)	3777 (74.6)	0.000
	Yes	57 (1.1)	30 (0.6)	
Past suicide attempts	No	1035 (20.5)	3561 (70.4)	0.000
	Yes	219 (4.3)	246 (4.9)	
Daily exposure to thin body ideal	No	674 (13.3)	2762 (54.6)	0.000
	Yes	580 (11.5)	1045 (20.6)	
Underweight	No	1182 (23.4)	3474 (68.6)	0.001
	Yes	72 (1.4)	333 (6.6)	

The findings of the chi‐square analysis and the multivariate analysis are presented in Tables 7 and 8, respectively. Participants with T1DM were 3.654 times more likely to be at risk of having EDs. Individuals who have a history of self‐reported schizophrenia may exhibit an increased susceptibility to develop (*p* = 3.582). In addition, individuals exhibiting the following self‐reported features demonstrated a heightened susceptibility to the development of EDs: female gender, IBD, daily exposure to a thin body ideal, past suicide attempt, borderline personality, sleep disorders, autism, food insecurities as well as being classified as underweight. Regular sports practice and weight satisfaction were associated with a decreased likelihood of developing EDs.

**TABLE 8:** Multivariate logistic regression.

Variables	Category	OR	95% CI		*p* value
			Lower	Upper	
Gender	Female	1.321	1.122	1.557	10^−3^
	Male				
Regular sport practice	Yes	0.480	0.404	0.571	10^−5^
	No				
T1DM	Yes	3.654	2.375	5.621	10^−5^
	No				
IBD	Yes	2.455	2.018	2.987	10^−5^
	No				
Do your parents push you to eat?	Yes	1.572	1.345	1.838	10^−5^
	No				
Schizophrenia	Yes	3.582	1.733	7.408	10^−3^
	No				
Borderline personality	Yes	1.836	1.161	2.903	10^−2^
	No				
Weight satisfaction	Yes	0.639	0.540	0.755	10^−5^
	No				
Food insecurities	Yes	1.544	1.302	1.830	10^−5^
	No				
Sleep disorders	Yes	1.307	1.066	1.601	10^−2^
	No				
Autism	Yes	2.331	1.377	3.947	10^−3^
	No				
Past suicide attempts	Yes	1.590	1.258	2.009	10^−4^
	No				
Daily exposure to thin body ideal	Yes	1.898	1.625	2.217	10^−5^
	No				
Underweight	Yes	1.620	1.192	2.203	10^−2^
	No				

## 4 Discussion

To the best of our knowledge, this is the first and largest multinational cross‐sectional study to conduct a screening of medical students in the MENA region regarding their potential risk of developing EDs through the EAT‐26 (Garner et al. 1982). Furthermore, 24.8% of students exhibited a high susceptibility to EDs. A history of self‐reported Type 1 diabetes, schizophrenia, IBD, and autism spectrum disorder (ASD) has been identified as being significantly associated with possible disordered food attitudes.

### 4.1 Screening of Medical Students at Risk of EDs

In 2019, Jahrami et al. conducted a global systematic review and meta‐analysis among medical students across nine countries with the aim of estimating the prevalence of at‐risk individuals of EDs (*N* = 5722). The included studies used screening tools such as the EAT‐26, SCOFF questionnaire (Sick, Control, One, Fat, Food), and eating disorder inventory (EDI). The overall rate was 10.4% (95% CI 7.8‐13.0%). Nonetheless, Pakistan was the only common country between our study and theirs. A comparable rate was reported by our study (26%) compared to the three Pakistani included studies: 21.7% (Babar et al. in 2002), 22.75% (Memon et al. in 2012), and 22.6% (Haroon et al. in 2016) (Jahrami et al. 2019). Furthermore, Fekih‐Romdhane et al. conducted, in 2022, a systematic review and meta‐analysis among medical students from nine countries (*N* = 21,383). The study included 5 countries from the MENA region (Egypt, Morocco, Saudi Arabia, Lebanon, and Palestine). The used screening tools were EAT‐26, SCOFF, and the eating disorder examination questionnaire (EDE‐Q) (Morgan et al. 1999; Garner et al. 1983). The overall rate of at‐risk individuals was 17.35% which is slightly lower than our rate. Analysis per country showed a rate of 12.11% in Saudi Arabia, which is also lower than our rate. Palestine had 25.96 % of at‐risk individuals which is higher than our findings (20%) (Fekih‐Romdhane et al. 2022). Our findings were consistent with other studies in the region. In Egypt, a study conducted at Tanta University among 615 medical students using EAT‐26 questionnaire demonstrated a prevalence of at‐risk individuals of 33.01% (Abo Ali and Shehata 2020). In Lebanon, two studies on medical students that used the EAT‐26 and SCOFF questionnaires, with sample sizes of 131 and 627, reported diverse rates: 16.79% and 30.14%, respectively (Bizri et al. 2020; Farchakh et al. 2019). In Palestine, a study on 1047 medical students using the EAT‐26 and SCOFF questionnaires as screening tools showed a rate of at‐risk of 21.11% (Damiri et al. 2021). While these studies shared similar population characteristics (age, sex, and BMI) and used the same screening tools, a huge disparity in the reported rate of at‐risk individuals is also documented (Fekih‐Romdhane et al. 2022).

Studies conducted in other developing countries reported different rates of EDs among medical students. In Malaysia, a study that included 1017 participants showed a prevalence of 13.86%. In India, two studies with sample sizes of 332 and 172 found prevalences of 15.06% and 16.86%, respectively. In Thailand, the prevalence was 15.93%. Despite using similar study characteristics and measurement tools, these global studies reported substantially lower prevalence rates compared to ours. Medical students from non‐Western cultures were shown to have a higher prevalence of ED risk as reported by Fekih‐Romdhane et al. 20.97% versus 12.98%. These differences could be explained by the generally tougher economic circumstances confronted by medical students in the MENA region compared to their counterparts in other countries (Fekih‐Romdhane et al. 2022; Iyer and Shriraam 2021; Garner et al. 1982; Chan et al. 2020; Ramaiah 2015). In light of ongoing globalization and modernization, there has been a notable increase in the incidence of ED symptoms and EDs within the Arab world. Furthermore, the rising prevalence of obesity, attributed to modern dietary habits, in turn, has led to various unhealthy behaviors such as dieting, compensatory behaviors, body dissatisfaction, and binge eating episodes. Significant sociocultural shifts have been observed, transitioning from a curvy body —historically viewed as a symbol of fertility and wealth—to that of a thin body. Thinner body aspiration has been associated with compensatory behaviors, binge eating, dieting practices, and heightened levels of body dissatisfaction (Melisse et al. 2020).

### 4.2 Type 1 Diabetes Mellitus

Individuals with self‐reported Type 1 DM have 2.47 times increased risk of developing EDs specifically BN and BED. A higher risk of insulin omission and misuse was reported particularly among females (Dean et al. 2024).

### 4.3 Inflammatory Bowel Disease

Our findings suggest an association between self‐reported IBD and disordered eating behaviors which is consistent with the literature. A study conducted by Satherley et al. reported that the prevalence of disordered eating behaviors is greater in populations with gastrointestinal (GI) disorders compared to healthy controls (Satherley et al. 2015). Disrupted behaviors may occur including corticoid refusal, medication abandonment, and/or intentional exacerbation of IBD symptoms (Ilzarbe et al. 2017).

### 4.4 Psychiatric Comorbidities

In our studied population, a self‐reported history of schizophrenia was associated with EDs which aligns with the literature. In fact, schizophrenia increases the risk of disordered eating behaviors, especially among females, due to factors such as the effects of antipsychotics, negative symptoms affecting appetite, and cognitive impairments that influence food intake and body image perception (Sankaranarayanan et al. 2021; Kouidrat et al. 2014; Malaspina et al. 2019). In addition, we found an association between a history of suicide attempts and the occurrence of disordered eating among our participants. These findings align with the results of the systematic review study conducted by Conti et al., which concluded a significant association between BED and increased suicidal behaviors and suicidal ideation especially when occurring alongside other psychiatric disorders. In fact, these individuals suffer from emotional distress, trauma, or mental health issues, which in turn may lead to restricting food intake or engaging in binge‐eating behaviors (Conti et al. 2017). A study conducted by Longo et al. emphasized the concordance between clinician and researcher diagnoses of psychiatric comorbidities in patients with AN. A poor agreement was found, except for OCD and substance use disorder. Patients with comorbidities identified by researchers showed more severe eating and psychopathology. Consequently, rigorous assessments for psychiatric comorbidities in AN are needed to ensure accurate diagnosis and treatment planning (Longo et al. 2022).

### 4.5 Autism Spectrum Disorder

Autistic individuals may struggle with sensory conditions that affect their relationship with food. Certain textures, tastes, or smells of food may be overwhelming or aversive, leading to restricted or selective eating patterns. In addition to rigid eating patterns, which can evolve into more severe EDs, our findings (2.3 greater risk among individuals with self‐reported ASD) align with the literature. In 2019, a systematic review that included 17 studies revealed that autistic participants have a higher risk of EDs and this comorbidity is most commonly diagnosed in patients with AN (Nickel et al. 2019; Dell'osso et al. 2018; Carpita et al. 2022).

### 4.6 Food Insecurities

The association between food insecurities and binge eating was demonstrated through a meta‐analysis conducted in 2023 which showed that adults experiencing food insecurities had higher odds of engaging in binge eating and BED compared to those in the food‐secure group (OR = 1.66 95% CI [1.42;1.93]), which is comparable to our findings (OR = 1.54 95% CI [1.30;1.83]. As a matter of fact, uncertainty about food availability can lead to disordered eating patterns and impact body image and self‐esteem and therefore the onset of EDs (Abene et al. 2023).

### 4.7 Sleep Disorders

The results from our population regarding the detrimental effect of poor sleep on eating behaviors correlate with the literature. Furthermore, individuals suffering from EDs are prone to inadequate sleep habits, including reduced sleep duration or lighter and more fragmented sleep. As a result, their sleep efficiency is reduced by an average of 3.6%. In an average month, a patient with an ED sleeps approximately 630 min less compared to the general population (Degasperi et al. 2024).

### 4.8 Protective Factors

In our studied population, regular sports practice and weight satisfaction were identified as protective factors against the development of disordered eating behaviors. Engaging in regular physical activities not only promotes physical health but also fosters a positive body image and enhances self‐esteem, providing a healthy outlet for stress and reducing the risk of EDs (El Ghoch et al. 2013). Weight satisfaction diminishes the likelihood of engaging in harmful dieting practices and promotes a balanced approach to food and nutrition. Therefore, encouraging regular sports practice and fostering a culture of body positivity and weight satisfaction may be effective in preventing the onset of EDs and supporting overall mental and physical well‐being (Kitsantas et al. 2003; Coker and Abraham 2014; Rekkers et al. 2021; Preston and Ehrsson 2018).

### 4.9 Limitations of the Study

While our paper employed rigorous methods, several limitations were observed. The voluntary nature of participation may result in a sample that is not fully representative of all medical students in the MENA region, as those with a particular interest or experience in EDs may be more inclined to participate. Furthermore, not all countries from this region were represented due to a lack of data collectors. These limitations could impact the generalizability of the findings to the broader student population. In addition, reliance on self‐report measures via electronic means introduces the risk of response bias, where participants may misreport their experiences due to social desirability or misunderstanding of survey questions. The prevalence of participants with various comorbidities was evaluated using “yes or no” questions rather than relying on medical records, which may potentially impact the reliability of the findings. Moreover, the cross‐sectional design restricts our ability to establish causality between EDs and the associated factors, providing only a snapshot rather than longitudinal insights. In addition, while validated questionnaires were used, they may not fully capture the complexity of eating disorder symptoms, leading to misclassification or underestimation of prevalence rates. The use of mean substitution to deal with missing data also constitutes a limitation. Finally, although statistical adjustments were made for potential confounders, these factors may still influence the observed associations.

## 5 Conclusion

Medical students in the MENA region, similar to their counterparts globally, are at increased risk of developing ED. Various risk factors contribute to this situation, including female gender, dysfunctional parental behaviors, and food insecurities. Some comorbidities among these students such as diabetes mellitus, ASD, IBD, and psychological comorbidities aggravate the outcome and deepen further the burden. Nowadays, unrealistic standards of body image, mainly thin ideals, can have detrimental effects on their body perception, psychological well‐being, and self‐satisfaction. Nevertheless, daily sports practice and weight satisfaction mitigate the risk of developing disordered eating habits, thereby enhancing overall well‐being. Thus, global and inclusive strategies of screening, support, and prevention are crucial in order to encounter this condition. Further studies are also needed to address this issue and establish the causality between EDs and potential associated factors in order to develop efficient future plans.

## List of Collaborators

Safa Gismalla (Faculty of Medicine and Health Sciences at Omdurman Islamic University, Sudan)

Maazouz Bensalem (Faculty of Medicine, Laghouat University, Algeria)

Basma El‐sayed Mohammad Risha (Faculty of Medicine for Girls, Alazhar University, Cairo)

Malek Nabhan (Faculty of medicine University of Aleppo)

Aya Algroum (Faculty of Medicine at Hashemite University)

Marwa Farag Ferjani (Faculty of Medicine University of Benghazi)

Toka Rabea Elbastawisy Salem (Faculty of Medicine, University of Mansoura, Dakahlia, Egypt)

Safaa Farag Ferjani (Faculty of Medicine University of Benghazi)

Emadedin Yussef Najjar (Faculty of medicine, University of Aleppo, Aleppo, Syria)

Miral Mohammad Abu Shawali (Faculty of Medicine at Hashemite University, Jordan)

## Author Contributions

**Siwar Belhaj Salem**: conceptualization, methodology, writing original draft, writing, reviewing, editing, project administration and formal analysis. **Fatima Ezzahraa El Idrissi, Ahmed Fikry mohamed, Husam Khrais, Rawan Elwalid Jad, Umair Ul Haq, Jaafer Ammar Jouini, Ramla Mohamed Farah Roble, Ronahy Haidar, Fatma Zaouali, Abdullah Janem, Hossam Salameh**: writing original draft, reviewing and data curation. **Ruzan Jamaleddin, Alaa Mohamed Elsayed, Fidha Hussain, Yasmine Adel Mohammed, Abeer Hagali, Zeyad Yassin, Shahd Almansour, Farah Bahs as, Dana AlMahder**: data curation and writing original draft. **Muhammad Shoaib Khan**: supervision and ressources.

## Conflicts of Interest

The authors declare no conflicts of interest.

### Peer Review

The peer review history for this article is available at https://publons.com/publon/10.1002/brb3.70417

## Supporting information



Supporting Information

## Data Availability

Data supporting this study are included within the article and supporting materials.
