# Supplementary material for: Screening of the Risk of Eating Disorders Among Medical Students in the MENA region and Its Associated Factors: A Multinational Cross‐Sectional Study
Source: Brain Behav. 2025 Mar 18;15(3):e70417. doi: 10.1002/brb3.70417 (PMC11919777; doi:10.1002/brb3.70417)
Supplement: Supplementary file 1 — Supporting Information [file BRB3-15-e70417-s001.pdf]

The prevalence of eating disorders (EDs) among medical students in the MENA region and its associated factors: A multinational cross-sectional study

1) Do you agree to participate in this survey?

Yes

No

2) Which language do you prefer?

Arabic

English

3) Are you a medical student?

Yes

No

❖ **Sociodemographic features**

1) Birth date

2) What is your gender?

Male

Female

3) What is your country of residency?

4) What is your current academic status?

First year

Second year

Third year

Fourth year

Fifth year

Sixth year

Intern

Resident

5) Marital status

Married

Single

Divorced

Widowed

6) Do you smoke?

Yes

No

7) Do you drink alcohol?

Yes

No

8) Do you practice sports regularly?

Yes

No

❖Eating disorders symptoms

1) Have you ever been diagnosed with eating disorders?

Yes

No

2) What type of eating disorders do you have?

Anorexia nervosa

Bulimia nervosa

Binge eating disorder

Avoidant or restrictive food intake disorder

Other

3) How often do you experience symptoms related with your eating disorder?

Daily

Weekly

Monthly

Yearly

Rarely

Never

4) How severe are your symptoms?

Mild

Moderate

Severe

Very severe

❖ **Eating disorders risk factors**

1) Do you have a past history of Type 1 Diabetes Mellitus?

Yes

No

2) Do you have a past history of inflammatory gastrointestinal diseases?

Yes

No

3) Do you have a past history of autism?

Yes

No

4) Have you been diagnosed with sleeping disorders?

Yes

No

5) How many hours do you sleep at night?

Less than 7 hours

7 to 9 hours

More than 9 hours

6) Do your parents think that you are overweight?

Yes

No

7) Do your parents push you to eat?

Yes

No

8) Which psychological comorbidities do you have?

Post-traumatic stress disorder (PTSD)

Obsessive compulsive disorder (OCD)

Social anxiety disorder

Bipolar disorder

Schizophrenia

Major depressive disorder

Borderline personality disorder

Perfectionism

Obsession

Impulsiveness

None

9) Is there any history of suicide attempts on your part?

Yes

No

10) Are you satisfied about your weight?

Yes

No

11) How much are you exposed to 'thin body ideal'?

Daily

Weekly

Monthly

Yearly

Rarely

Never

12) Do you have any food insecurities? (The condition of not having access to sufficient food, or food of an adequate quality, to meet one's basic needs.)

Yes

No
